# The potency of mesenchymal stem/stromal cells: does donor sex matter?

**DOI:** 10.1186/s13287-024-03722-3

**Published:** 2024-04-22

**Authors:** Ghada Maged, Menna A. Abdelsamed, Hongjun Wang, Ahmed Lotfy

**Affiliations:** 1 Department of Biochemistry, Faculty of Science, Alexandria University, Alexandria, Egypt; 2 Biotechnology and Life Sciences Department, Faculty of Postgraduate studies for Advanced Sciences, Beni-Suef University, Beni Suef, Egypt; 3 Department of Surgery, Medical University of South Carolina, 29425 Charleston, SC USA; 4 Ralph H. Johnson Veterans Affairs Medical Center, Charleston, SC USA

**Keywords:** Mesenchymal stem/stromal cells, MSCs, Sex, Heterogeneity, Potency

## Abstract

Mesenchymal stem/stromal cells (MSCs) are a promising therapeutic tool in cell therapy and tissue engineering because of their multi-lineage differentiation capacity, immunomodulatory effects, and tissue protective potential. To achieve optimal results as a therapeutic tool, factors affecting MSC potency, including but not limited to cell source, donor age, and cell batch, have been investigated. Although the sex of the donor has been attributed as a potential factor that can influence MSC potency and efficacy, the impact of donor sex on MSC characteristics has not been carefully investigated. In this review, we summarize published studies demonstrating donor-sex-related MSC heterogeneity and emphasize the importance of disclosing donor sex as a key factor affecting MSC potency in cell therapy.

## Introduction

Mesenchymal stem/stromal cells (MSCs) are multipotent adult stem cells that can be obtained from various tissues, such as adipose tissue, bone marrow, umbilical cords, and other sources. MSCs are a popular source of cell therapy in regenerative medicine due to their multi-lineage differentiation capacity, immunomodulatory effects, and tissue protective potential [1]. In vitro, MSCs can proliferate and differentiate into various cell types, including adipocytes, osteoblasts, chondrocytes, and others. When infused in vivo, MSCs can replace damaged cells and tissues [2–4]. MSCs can also secrete growth factors, extracellular vesicles, and mitochondria that promote the survival of other cells through paracrine effects [5, 6]. These unique characteristics have attracted dramatic attention to MSCs as an efficient therapeutic tool [7].

To date, the therapeutic effects of MSCs have been tested in many animal models and more than 1,138 human clinical trials [8]. Even though promising results were observed in different preclinical animal disease models, most of the MSC clinical trials for various human diseases have not achieved their anticipated outcomes; this discrepancy could be attributed to inconsistent MSC criteria or, in other words, MSC heterogeneity [9].

MSCs exhibit biological heterogeneity based on several criteria, including donor age, source of donor tissue, as well as differences found in cell clones and batches. For example, MSCs derived from younger donors are more potent than those from elderly donors. Likewise, MSCs from separate sources such as bone marrow (BM-MSCs) or adipose tissue (ASCs) also differ in certain aspects, e.g., ASCs have a higher proliferation rate than BM-MSCs [10–13].

Due to the increasing demand for cell-based therapy, it is imperative to assess additional factors affecting the potency of MSCs. Donor sex has recently been recognized as a factor affecting MSC characterization, potency, and therapeutic efficiency. In this review, we discuss factors that contribute to MSC heterogeneity and potency with an emphasis on published studies describing the impact of donor sex on MSC proliferation, differentiation capabilities, gene expression, and therapeutic effects.

## MSC Heterogeneity

**Fig. 1:**
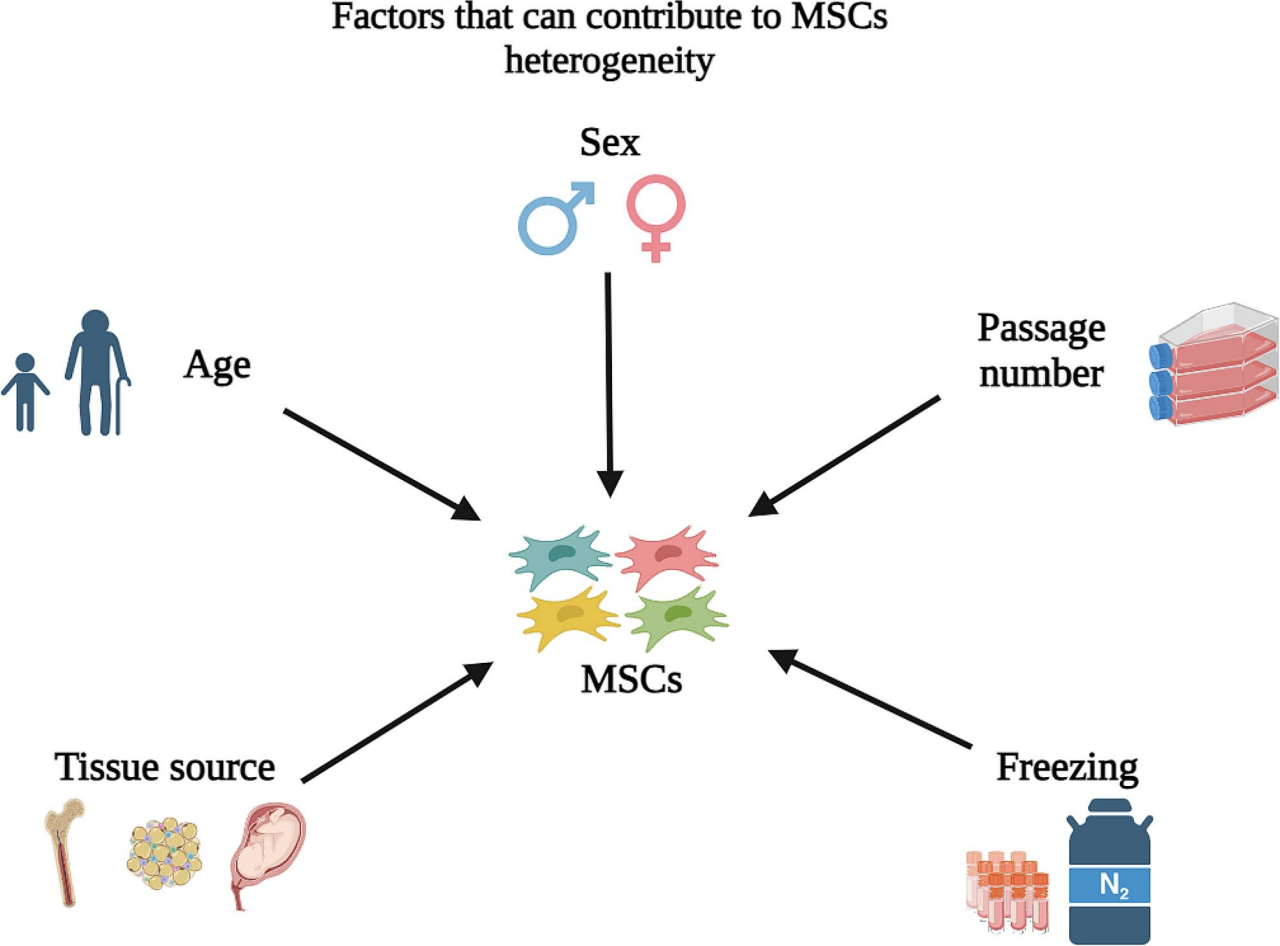
Factors that can contribute to MSCs heterogeneity

The heterogeneity of MSCs can arise from various donor-related factors, such as donor age and tissue sources, or non-donor-related factors, such as cell batch; passage; and freezing/thawing process (Fig. 1) [14–17]. Below are examples of how these factors could result in MSC heterogeneity and affect their potency when used in disease therapy.

First, donor age is considered a quintessential factor that should be considered when using MSCs in cell therapies. The potency of MSCs declines with age. For instance, ASCs from elderly human donors (> 60 years) displayed more senescent features with reduced differentiation potential and produced fewer colonies when compared to younger donors (< 30 years) [11]. Older age also showed a negative effect on the yield of BM-MSCs in rats, with the younger age (4 weeks) having the maximum yield of MSCs compared to 48-week-old rats [12]. Therefore, strategies such as licensing might be needed to enhance the yield, cell proliferation, and expansion capabilities of MSCs from aged donors if autologous cells are used [11]. A study by Li and colleagues showed human MSCs isolated from aged donors (65–80 years old) showed a lower expression of fibroblast growth factor 2 (FGF2) and a higher level of senescent activity than MSCs isolated from younger donors (18–25 years old) [13]. Another study also demonstrated a significant decline in the quantity of MSCs in bone marrow associated with older age due to the decrease in bone density [18].

The tissue source from which MSCs are derived can also contribute to their heterogeneity. Specifically, MSCs obtained from different tissues could exhibit variations in their differentiation potential, proliferation rate, immunomodulatory properties, and gene expression profiles [16, 19]. For instance, BM-MSCs and ASCs show greater penchants to differentiate into osteoblasts and better colony-forming abilities than umbilical cord-derived MSCs (UC-MSCs). On the other hand, UC-MSCs have a higher proliferation rate and a higher tendency toward chondrogenic differentiation than BM-MSCs and ASCs [20]. There is also heterogeneity between human ASCs and human BM-MSCs, as approximately 1,400 genes related to tenogenic potential and chemotaxis exhibited differences between these two types of cells. Lastly, although adequate chondrogenesis has been demonstrated by both dental pulp-derived MSCs (DPSCs) and periodontal ligament-derived MSCs, DPSCs uniquely show a higher tendency towards both osteogenesis and adipogenesis [21].

## The impact of donor sex on MSC heterogeneity and potency

Recent studies have revealed that the characteristics of MSCs could vary depending on the donor’s sex (summary of human MSC studies in Table 1 and animal MSC studies in Table 2). We discuss the differences between MSCs from male and female donors and how donor sex impacts proliferation, differentiation capability, gene expression, immunomodulatory and therapeutic effects, and other biological characteristics of MSCs.

**Table 1:** Summary of studies on human MSC heterogeneity caused by donor sex

MSCs source	Number of Donors	Aspect of differences	Ref.
Adipose tissue	N/A	Assessing transcriptomic profiles of ASCs derived from male and female donors using TRAM software unveiled donor-sex-related dimorphism, influencing chromosomal segments, some expressed genes, and potency variations. Also, results indicate that female ASCs are likely to differentiate into adipocytes compared to male ASCs.	[38]
Adipose tissue	M:7 F:7	Female ASCs exhibit superior immunosuppression potency compared to male ASCs in vitro, as they can consistently suppress PBMC proliferation more effectively.	[51]
Adipose tissue	M:3 F:3	Male ASCs have osteogenic differentiation more effectively than female ASCs.	[34]
umbilical cord	M:6 F:6	The expression of OCT4 and DNMT1 genes significantly elevated in UC- MSCs isolated from male, as compared to UC-MSCs isolated from female.	[40]
umbilical cord	M:5 F:5	Male fetal UC-MSCs displayed a significantly higher proliferation and adipogenic ability than female fetal UC-MSCs. Additionally, male MSCs are more potent in the inflammatory cytokines’ expression to LPS-induced inflammation.	[26]
Bone Marrow	M:28 F:25	Female BM-MSCs exhibited a significantly greater ability to suppress T cell proliferation compared to males. BM-MSCs obtained from younger female donors displayed high clonogenic potential, faster division rates, and increased frequency.	[25]
Bone Marrow	M:7 F:12	Breast cancer cells proliferate increasingly when MSCs from female donors are involved.	[31]
Bone Marrow	M:6 F:3	Female BM-MSCs’ sphingolipidome consisted of 88.35% ceramide, and 10.18% sphingomyelin. On the other hand, male BM-MSCs’ sphingolipidome included 54% ceramide, and 44.53% sphingomyelin. This difference could be associated with different influences on the cell properties.	[60]
Bone Marrow	M:26 F:32	BM-MSCs collected from osteoporotic females had more probability to exhibit an enhanced differentiation potency toward adipocytes formation; considered a non-desired differentiation outcome for bone regeneration.	[36]

Abbreviations: M: Male, F: female, NA: data not available, BM-MSCs: Bone marrow mesenchymal stem cells, ASCs: Adipose tissue derived stem cells, PBMCs: Peripheral blood mononuclear cells, UC-MSCs: Umbilical cord-derived MSCs, LPS: lipopolysaccharide, TRAM: transcriptome mapper

**Table 2:** Summary of studies on animal MSC heterogeneity caused by donor sex

MSCs source	Species	Number of Donors	Aspect of differences	Ref.
Bone Marrow	Rat	N/A	The estrogen mitogenic effects were more pronounced in female BM-MSCs, however, combinations of estrogen and dexamethasone is more effective in promoting male BM-MSCs proliferation with different steroid optimal doses and interactions based on donor gender.	[29]
Bone Marrow	Rat	N/A	Therapeutically, female BM-MSCs have greater efficacy than male MSCs in reducing neonatal hyperoxia-induced lung inflammation and vascular remodeling. The beneficial effects of female MSCs were more pronounced in male animals.	[56]
Bone Marrow	Rat	N/A	Female BM-MSCs provided a greater protection of myocardial function than their male-derived. The Bcl-xl/Bax ratio is significantly increased after the treatment with BM-MSCs from female donors than their male counterparts	[57]
Bone Marrow	Rat	M:6 F:6	Male bone marrow contained significantly higher BM-MSCs than female rats, represented by high CFU numbers in both femora and tibiae	[59]
Bone Marrow	Mouse	M:4 F:4	BM-MSCs from female mice have lower osteogenesis than cells from male littermates.	[35]
Bone marrow	Monkey	M:6 F:6	Upon differentiation, female BM-MSCs acquire higher neurogenic potential compared with male BM-MSCs.	[33]
Bone Marrow	pig	M:3 F:3	Supplementation of beta- estradiol resulted in anti-apoptotic activity up-regulation in only female BM-MSCs, but not in male BM-MSCs.	[30]
Adipose tissue/ Dermal Skin	Pig	M:3 F:3	Female ASCs were found to be more resistant to senescence under in vitro culture conditions.	[27]

Abbreviations: M: Male, F: female, NA: data not available, BM-MSCs: Bone marrow mesenchymal stem cells, ASCs: Adipose tissue derived stem cells, PBMCs: Peripheral blood mononuclear cell, UC-MSCs: Umbilical cord-derived MSCs.

### MSC Proliferation

One of the MSCs features is the in vitro proliferation [1, 22–24]. Among MSCs from different sources, BM-MSCs have been found to divide more rapidly when taken from younger females compared to those from males [25]. On the other hand, human UC-MSCs isolated from heterosexual twins showed that male fetal UC-MSCs had a significantly higher proliferation capacity than female fetal UC-MSCs, which has been attributed to higher expression levels of *NANOG, TERT, OCT4,* and *SOX2* in UC-MSCs [26]. Moreover, assessing the gender–related characteristics in ASCs has also proven the gender-specific heterogeneity in MSC biology [27, 28]. For example, ASCs from female donors show a greater ability to maintain their proliferative capacity in vitro than their male counterparts due to the higher expression of the OCT3/4 protein, a transcription factor indicative of the proliferative capacity of MSCs [27].

In a study of the steroid effect on BM-MSCs proliferation, it was reported that the optimal dose and interaction of steroids varied depending on donor sex. For instance, the mitogenic effects of estrogen on rat MSCs showed more pronounced effects in females with a concentration of 10 ^− 10^ M-10 ^− 12^ M 17β-estradiol (E2). However, combinations of estrogen and dexamethasone were more effective in promoting male rat MSC proliferation [29]. On the contrary, the optimal dose of E2 for the proliferation capacity of BM-MSCs derived from male and female mini-pigs was similar in both sexes and equal to 10 ^− 12^ M [30]. Nonetheless, faster proliferation was observed in MSCs isolated from female rats than in male MSCs cultured in conventional or steroid-free media [29]. However, while female BM-MSCs have been shown to have beneficial properties, their use may contribute to an increase in the proliferation of breast cancer cells when cultured together. Therefore, caution should be exercised when considering the use of BM-MSCs in breast cancer therapy [31].

### MSC differentiation capabilities

In vitro differentiation is one of the defining features of MSCs and can be measured by their ability to differentiate into osteogenic, chondrogenic, and adipogenic lineages under specific circumstances [4]. In addition, MSCs can differentiate into other cell types, such as skeletal myocytes and tenocytes, with the appropriate environmental cues [32]. Studies have been conducted to compare the gender-related differences in the MSC differentiation. A prime example is a study conducted on BM-MSCs which demonstrated that neither sex nor donor age affected the in vitro mesodermal differentiation capacity of BM-MSCs [25]. This study analyzed BM-MSC adipogenesis, osteogenesis, and chondrogenesis abilities respectively. No significant differences related to donor age or sex were observed [25]. On the other hand, a higher potential capacity for neurogenic differentiation at passage 10 was notable with an elevation of γ-aminobutyric acid (GABA) synthesis and release with female rhesus monkey BM-MSCs in comparison to nestin-positive male BM-MSCs due to a higher production of nestin-positive cells observed in the female BM-MSCs [33].

Under suitable conditions, ASCs exhibit the ability to undergo osteogenesis. Human ASCs from males and females were isolated from superficial and deep fatty layers of the abdominoplasty specimens and were cultured in osteogenic media. Markers for osteogenesis and their relations with sex were evaluated 1, 2, and 4 weeks after differentiation induction. Results showed a significant difference in the differentiation efficiency between male and female ASCs from both superficial and deep depots, with a higher degree in males than females. Furthermore, superficial male depot ASCs displayed faster and more efficient differentiation than their deep counterparts. On the contrary, the osteogenic differentiation degree was not significantly different between female ASCs from superficial or deep depots [34]. Moreover, in a mouse study, BM-MSCs from female mice showed lower osteogenesis than cells from male littermates [35].

Successful bone regeneration depends on various factors, with steroids functioning as an effective modulator regulating osteogenic differentiation. The regulatory effect of steroids is determined by the dose required for osteogenic markers up-regulation, which is sex-dependent. A higher activity of alkaline phosphatase (ALP), an early marker of osteogenic differentiation, was observed in female BM-MSCs treated with lower concentrations of E2, but not in male BM-MSCs [29]. In another study, the enrichment of BM-MSCs isolated from osteoporotic female donors with ALP and PDGFRα^+^/CD146^-^/CD362^-^ cells had a greater than 50% likelihood of exhibiting increased differentiation capacity towards adipocyte formation, which is an undesirable outcome for bone tissue regeneration [36]. Nonetheless, Non-osteoporotic male donors who received vitamin D supplementation and had an enriched population of CD146^+^/ALP^+^/CD14^-^ cells showed a more than 50% increase in their osteoblast differentiation capacity. Hence, non-osteoporotic males were more suitable for enhancing in vitro mineralized matrix formation than females [36]. On the other hand, the mature osteoblastic marker, osteocalcin, showed similar peak levels in both males and females. It suggests that the sex differences during osteogenic differentiation with E2 supplementation may be contributed to the variation in steroid receptors [29].

### MSC Gene expression

Although male and female genomes are nearly identical, there are differences at the molecular level due to variations in gene expression [37]. Different chromosomic segments and gene expressions were also identified in human ASCs isolated from male and female donors by the transcriptome mapper (TRAM) meta-analysis. This finding resulted in a hypothesis that ASC characteristics such as differentiation, proliferation, modulation, and senescence may vary because of the donor sex of ASCs. Indeed, variations of expressions of inflammation-related genes including C-X-C motif ligands and immunoglobulin (e.g., Immunoglobulin Lambda Constant (*IGLC*) 1 and 3)) contributed to the variations in the immune modulatory capacity ASCs derived from male and female donors. While C-X-C motif ligands were expressed at a low level in males, *IGLC1* and *IGLJ3* (immunoglobulin lambda joining 3) were highly expressed compared to ASCs derived from females [38].

Previous studies have shown the integral role of CXCL3 in promoting adipogenic differentiation in mouse preadipocyte and MSC cell lines [39]. TRAM results indicate that female ASCs are likely to differentiate into adipocytes compared to male ASCs because of low abundance of CXCL3 in male cells [38]. In addition, stem cell proliferation and migration may differ between cells from each gender because of variations in the expression of cell cycle regulators such as *TFPI2, GNG11, ANKK1*, and *CAMTA1*.

Indoleamine 2, 3, dioxygenase (IDO) is critical for the immunosuppressive function of MSCs. Female BM-MSCs expressed higher levels of *IDO1* compared to male BM-MSCs, suggesting a better capacity to suppress the proliferation of T cells [25]. On the other hand, there was no correlation between the expression of *Oct4, Nanog, and Prdm14* mRNA and the donor’s sex or age in BM-MSCs [25]. However, the expression of the stemness-regulating gene *Oct4* significantly differed between male and female MSCs derived from Wharton’s jelly [40]. Upregulation of *Oct4 *is associated with the upregulation of DNMT1. This methyltransferase plays a critical role in maintaining methylation patterns during DNA replication [41], with higher expression in males indicating a sex-dependent epigenetic modulation [40]. [].

MSCs secrete vascular endothelial growth factor A (VEGF-A) and transforming growth factor-β (TGF-β), which are both linked to in vitro and in vivo angiogenesis and anti-fibrotic processes [42]. A previous study revealed a notable difference in gender-based *VEGF-A* and *TGF-β* gene expression with a significant upregulation in male UC-MSCs compared to female UC-MSCs. As a result of this, it is reasonable to predict that male UC-MSCs may have better angiogenesis and anti-fibrotic processes potential than female UC-MSCs [26].

### Immunomodulatory effect of MSCs

MSCs exert immunomodulatory and immunosuppressive effects through various mechanisms. They interact with immune cells such as T cells, B cells, and natural killer cells, modulating immune responses [43, 44]. The immunomodulatory properties are attributed to the production of metabolites, cytokines, and growth factors by MSCs. The immune suppressive potential of MSCs involves cell-to-cell contact and the secretion of immune regulatory molecules [44–46].

The immunosuppressive properties of MSCs can also be manifested via paracrine effects of cell communication, cell adhesion molecules, and extracellular vesicles (EVs) [47, 48]. For instance, in tissue injury caused by autoreactive T cells in autoimmune diseases, this type of cell communication is recruited to evoke immune response and EVs are carried to the site of inflammation by biofluids to modulate immunity [49]. MSCs have also been shown to produce several immunomodulatory factors including IL-10, IL-1 receptor antagonist (IL-1Ra), TGF-β and the cell adhesive molecules intercellular adhesion molecule-1 (ICAM-1) and vascular cell adhesion molecule 1 (VCAM-1) [48, 50] that mediate their immunosuppressive properties.

The impact of donor sex on the functionality and potency of MSCs, especially their immunomodulatory function, has been recently investigated. An in vitro study of ASCs from male and female human donors has shown that ASCs-mediated immunomodulation is likely donor-sex-specific [51]. Female ASCs produced significantly higher concentrations of the anti-inflammatory mediators IL-1Ra, PGE-2, and IDO than male ASCs. In addition, female ASCs showed prolonged expression of the adhesive molecule VCAM1 when compared to male ASCs. Female ASCs also promoted the downregulation of IL-2 receptor gene expression in PBMCs, leading to higher immunosuppression levels in comparison to male ASCs [51].

Sex-related immunosuppressive properties were also assessed in human BM-MSCs [25]. Interferon- γ receptor 1 (IFN-γR1) and IL-6β expression in female BM-MSCs were higher than their male counterparts. mRNA analysis has revealed a higher *IDO1* mRNA expression in female than male BM-MSCs. In addition, female BM-MSCs exhibited a better immunosuppressive ability against T cell proliferation than male BM-MSCs. This suppression is suggested to be mediated by IDO1 [25]. On the contrary, a study by Zhang et al., has shown that there are no sex-related differences in immunosuppressive properties in UC-MSCs from heterosexual twins [26].

### Therapeutic effect of MSCs

MSCs have therapeutic effects against many diseases because of their ability to regenerate and repair damaged tissues [4]. It was reported that multiple factors including donor sex affect the therapeutic efficacy of MSCs [52].

For instance, a study reported that the therapeutic efficacy of muscle-derived MSCs (MDSCs) for skeletal muscle regeneration is donor-sex dependent. Specifically, the regeneration potential of skeletal muscles was compared in vivo by transplanting male or female MDSCs into dystrophic mice. Subsequently, the data showed that female MDSCs could regenerate skeletal muscles more efficiently than their male counterparts [53].

It has also been evidenced that the chondrogenic and osteogenic differentiation potential of human MDSCs in bone and cartilage regeneration is donor sex-related [54]. Human MDSCs from male donors have been found to exhibit more chondrogenic and osteogenic potential than MDSCs from female donors in vitro. The in vivo tests of the two genders derived MDSCs further revealed that male MDSCs were more efficient in bone regeneration. Micro computed tomography (MicroCT) test showed more bone regeneration at 2 weeks with a higher bone density at 4 and 6 weeks after transplantation of male MDSCs compared to their female counterparts [54]. In contrast, no difference was observed between male and female donor MSCs when their therapeutic efficacies in recovering bone loss were compared in the gonadectomy mouse model in different recipient genders [55].

MSCs have presented therapeutic efficacy in neonatal hyperoxia-induced lung injury. Ibrahim Sammour and colleagues studied the effect of BM-MSC donor sex on the therapeutic potential of neonatal hyperoxia-induced lung injury [56]. The study revealed that BM-MSCs from female donors have a higher therapeutic potency than their male counterparts in attenuating inflammation in neonatal hyperoxia-induced lung injury. Female BM-MSCs are also superior to male MSCs in improving vascular remodeling [56]. Interestingly, the beneficial effect of the female MSCs is more preferential when given to male recipients [56].

Female MSCs have also been shown to exhibit a higher protective advantage than their male counterparts in sepsis and endotoxemia [57]. Manukyan et al. have examined the impact of donor sex on the therapeutic potential of MSCs in an endotoxemic cardiac dysfunction model in adult male Sprague-Dawley rats. By analyzing the myocardial functions of the injected rats, they found that female MSCs provided better protection of myocardial function than male MSCs [57]. In addition, the Bcl-xl/Bax ratio, an indicator for cell survival, is significantly increased after the treatment with female than male MSCs, suggesting a larger therapeutic potential of female MSCs than male MSCs against acute endotoxemic injury.

All these together underline that donor sex is a contributing factor affecting the therapeutic potential and potency of MSCs. The question is: “What factors determine the donor sex-related therapeutic potential?” Investigators argue that these reasons might be found in sex-related innate factors which are differentially expressed in male and female MSCs. The role of the sex-related hormone estrogen in providing the advantage of female MDSCs over male MDSCs in skeletal muscle regeneration has been tested. However, neither pre-stimulation of the male MDSCs with estrogen nor the transplantation of male MDSCs into female recipients improved the rate of skeletal muscle regeneration [53]. Male and female MDSCs were suggested to be able to respond to stress through different cellular pathways [53]. When MDSCs were exposed to oxidative stress, male MDSCs showed an increased differentiation rate while female MDSCs maintained a lower proliferation rate. Male MDSCs may have been depleted rapidly in the transplantation site, and female MDSCs would have a higher tissue regeneration ability than their male counterparts [53].

### Other biological characteristics of MSCs

There are additional biological characteristics that expressed differences between male and female MSCs, including but not limited to cellular markers, cell senescence, sphingolipids levels, and even MSC quantities.

MSCs are heterogeneous cells with subpopulations that may influence their proliferative, pluripotent, and apoptotic features. Concerning donor sex, it has been revealed that diverse subpopulations may vary between cells from males and females. The pro and anti-inflammatory cytokine IL-6 has a higher expression in male than in female ASCs [27]. Moreover, there was a higher expression of the senescence-associated β-galactosidase (SA-β-Gal), a cell aging marker, in male ASCs than in their female counterpart [27]. The sex differences in cellular senescence have been reported in many other cell types and the causes are not clear yet; however, some factors such as sex chromosomes may play a role [58].

Interestingly, the quantity could differ between male and female MSCs. Strube P et al. showed that male rat bone marrow contained significantly higher BM-MSCs than female rats, represented by high colony-forming unit numbers in both femora and tibiae [59].

Assessing the profile of sphingolipids in BM-MSCs derived from different genders using liquid chromatography/tandem mass spectrometry revealed sex-related heterogeneity which may have contributed to differences in potency [60]. Male BM-MSCs have higher ratios of sphingomyelin, hexosylceramide and long-chain bases (LCBs) than their female counterparts (44.53%, 1,48% and 1.48% vs. 10.18%, 0.68% and 0.61%, respectively) whereas female BM-MSCs have a higher percentage of ceramides than their male counterparts (88.35% vs. 54%, respectively) [60]. The LCB profile of male BM-MSCs also differs from female BM-MSCs. In male BM-MSCs, LCBs consist of 23.64% sphingosine (Sph), 8.04% sphingosine-1- phosphate (S1P), 28.07% sphinganine (Sa), 6.18% sphingosine-1 phosphate (Sa1P), 21.84% glucosyl sphingosine (GlcSph) and 12.22% lysosphingomyelin (LSM). However, female BM-MSCs LCBs consist of 76% Sph, 4.59% S1P, 16.69% Sa, 2.57% Sa1P, and 0.15% LSM. These changes in LCB profile have its impact on cell’s biological functions. For instance, the increase in the S1P enhanced the therapeutic efficacy of MSCs in pulmonary arterial animal model hypertension [61].

## Further perspective

The influence of MSC donor sex in MSCs heterogeneity and potency has yet to be carefully investigated. This review sheds light on the significance of considering the MSC donor sex-related characteristics that might give MSCs derived from one gender an advantage over the other as a therapeutic tool.

**Fig. 2:**
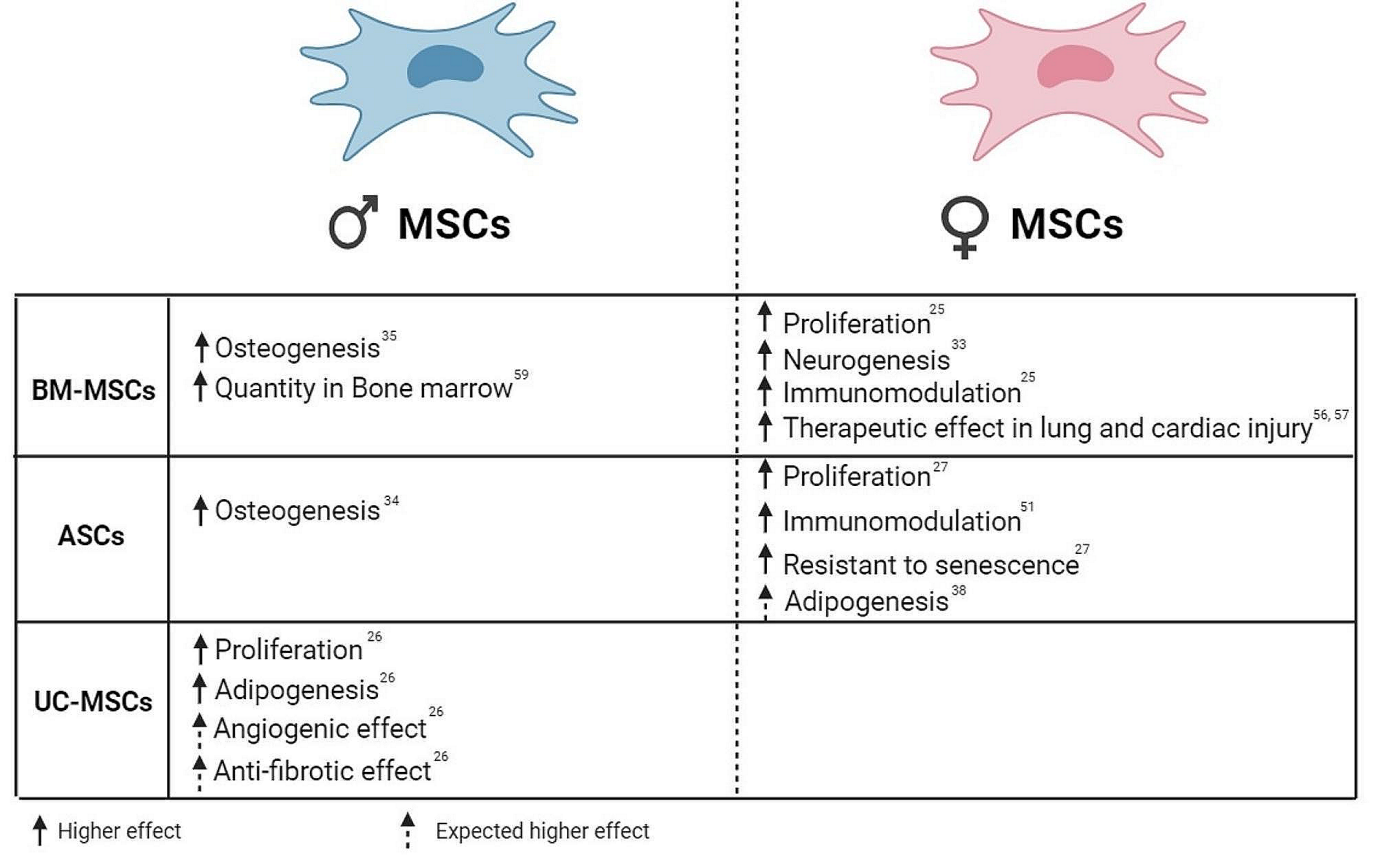
Illustration of the main differences between male and female MSCs.

Defining these mechanisms of how MSCs from specific donor sex can differ from the other will uncover factors which can influence the therapy outcomes. Furthermore, some autoimmune diseases such as systemic lupus erythematosus, multiple sclerosis, Sjogren’s syndrome, Grave’s disease, and Hashimoto’s thyroiditis, are prevalent in females. HIV infection also showed genetic disparities between males and females, and unequally affects women more than men [62, 63]. Considering the encouraging results of MSCs and their clinical application in treating these conditions, we propose investigators report as many donor characteristics as possible, especially donor sex, that might contribute to the study outcomes when publishing their results. MSCs from a specific sex may have more therapeutic effects for these sex-biased diseases for a common reason. Consequently, since there are limited studies regarding the impact of sex on MSC-based cell therapy outcomes in these diseases, further investigations are necessary to optimize MSCs-based therapy. These studies will provide valuable insights for enhancing the treatment outcomes. As a result, after expanding our understanding of the interplay between donor or recipient sex and diseases and its significant impact on treatment results, the tailoring of cell therapies for diseases would become sex specific. In addition, to ascertain the sex specific MSCs safety and feasibility in diseases, it is essential to consider appropriate environmental cues in conjunction with sex to achieve the desired clinical outcomes. For example, female BM-MSCs and ASCs showed higher immunomodulation effects than male BM-MSCs and ASCs, which suggests that female BM-MSCs/ASCs are likely superior in treating autoimmune diseases than male BM-MSCs/ASCs (Fig. 2). On the other hand, male BM-MSCs and ASCs may have a greater osteogenic potential, suggesting they have better effect in treating bone disorders. Moreover, female BM-MSCs showed greater therapeutic potential against lung and cardiac injury than male counterparts in animal models, suggesting that female BM-MSCs would better treat lung or cardiac injuries (Fig. 2). Accordingly, it seems in the future, it will be beneficial to determine the MSC donor sex depending on the targeting disease.

## Conclusion

Over the last few decades, MSCs have been tested as a therapeutic tool in treating various diseases. However, their therapeutic effect varied mainly due to their heterogeneity. Although some studies listed in this review were only done with limited donors, they strongly suggest that donor sex is an important factor that contributes to MSC heterogeneity and potency, and emphasize the importance of considering donor sex when preparing MSC for animal studies or clinical trials to achieve optional therapeutic effects.

## Data Availability

Not applicable.

## Abbreviations

MSCs: Mesenchymal stem/stromal cells
BM-MSC: Bone marrow mesenchymal stem cells
ASC: Adipose tissue derived stem cells
PBMC: Peripheral blood mononuclear cell
UC-MSCs: Umbilical cord-derived MSCs
DPSCs: Dental pulp-derived MSCs
LPS: Lipopolysaccharide
TRAM: Transcriptome mapper
E2: β-estradiol
FGF2: Fibroblast growth factor 2
IDO: Indoleamine 2, 3, dioxygenase
ALP: Alkaline phosphatase
Sph: Sphingosine
S1P: Sphingosine-1-phosphate
Sa: Sphinganine
Sa1P: Sphingosine-1 phosphate
GlcSph: Glucosyl sphingosine
LSM: lysosphingomyelin
